# Catching the Right Wave: Evaluating Wave Energy Resources and Potential Compatibility with Existing Marine and Coastal Uses

**DOI:** 10.1371/journal.pone.0047598

**Published:** 2012-11-07

**Authors:** Choong-Ki Kim, Jodie E. Toft, Michael Papenfus, Gregory Verutes, Anne D. Guerry, Marry H. Ruckelshaus, Katie K. Arkema, Gregory Guannel, Spencer A. Wood, Joanna R. Bernhardt, Heather Tallis, Mark L. Plummer, Benjamin S. Halpern, Malin L. Pinsky, Michael W. Beck, Francis Chan, Kai M. A. Chan, Phil S. Levin, Stephen Polasky

**Affiliations:** 1 The Natural Capital Project, Stanford University, Stanford, California, United States of America; 2 Ocean Science and Technology Institute, Inha University, Nam-gu, Incheon, Korea; 3 Western Ecology Division, U.S. Environmental Protection Agency, Corvallis, Oregon, United States of America; 4 Biodiversity Research Centre, University of British Columbia, Vancouver, British Columbia, Canada; 5 NOAA Northwest Fisheries Science Center, Seattle, Washington, United States of America; 6 National Center for Ecological Analysis and Synthesis, Santa Barbara, California, United States of America; 7 Department of Ecology and Evolutionary Biology, Princeton University, Princeton, New Jersey, United States of America; 8 Global Marine Team, The Nature Conservancy, Santa Cruz, California, United States of America; 9 Department of Zoology, Oregon State University, Corvallis, Oregon, United States of America; 10 IRES, University of British Columbia, Vancouver, British Columbia, Canada; 11 Department of Applied Economics, University of Minnesota, St. Paul, Minnesota, United States of America; Plymouth University, United Kingdom

## Abstract

Many hope that ocean waves will be a source for clean, safe, reliable and affordable energy, yet wave energy conversion facilities may affect marine ecosystems through a variety of mechanisms, including competition with other human uses. We developed a decision-support tool to assist siting wave energy facilities, which allows the user to balance the need for profitability of the facilities with the need to minimize conflicts with other ocean uses. Our wave energy model quantifies harvestable wave energy and evaluates the net present value (*NPV*) of a wave energy facility based on a capital investment analysis. The model has a flexible framework and can be easily applied to wave energy projects at local, regional, and global scales. We applied the model and compatibility analysis on the west coast of Vancouver Island, British Columbia, Canada to provide information for ongoing marine spatial planning, including potential wave energy projects. In particular, we conducted a spatial overlap analysis with a variety of existing uses and ecological characteristics, and a quantitative compatibility analysis with commercial fisheries data. We found that wave power and harvestable wave energy gradually increase offshore as wave conditions intensify. However, areas with high economic potential for wave energy facilities were closer to cable landing points because of the cost of bringing energy ashore and thus in nearshore areas that support a number of different human uses. We show that the maximum combined economic benefit from wave energy and other uses is likely to be realized if wave energy facilities are sited in areas that maximize wave energy *NPV* and minimize conflict with existing ocean uses. Our tools will help decision-makers explore alternative locations for wave energy facilities by mapping expected wave energy *NPV* and helping to identify sites that provide maximal returns yet avoid spatial competition with existing ocean uses.

## Introduction

Wave energy has the potential to generate substantial amounts of clean, safe, reliable, affordable and renewable electricity, thereby making it an appealing way to meet burgeoning energy demands [1]. Although in its infancy, the wave energy industry may be poised to grow as rapidly as the offshore wind industry, which has become established in several northern European countries in the past decade. Among various renewable energy resources (e.g., solar, wind, and tidal energy), wave energy has the highest power density and provides relatively continuous and predictable power, which is advantageous for electrical grid operation [2]. Costs of electricity generated by wave energy have decreased since the 1980s and are likely to decrease further as technologies develop and the industry expands [3]. As the costs of energy from fossil fuels increase, wave energy may become economically feasible in the near future. Consequently, decision-makers, the private sector and the public are interested in converting wave energy into electricity. Two important steps in this process are evaluating a site’s capacity to produce electricity and identifying potential impacts on the surrounding ecosystem and the activities it supports [4].

While waves may provide a source of clean and renewable energy, wave energy conversion projects may conflict with existing ocean uses or strategies for protecting marine species and habitats. The potential impacts of wave energy conversion facilities include changes in fishing opportunities, pelagic and benthic habitat, recreational activities, aesthetic views, hydrodynamic and wave environments, and navigational hazards [5], [6], [7]. Many of the potential impacts are site-specific and the magnitude of these impacts on coastal and marine ecosystems is poorly understood because of the as-yet limited experience with wave energy conversion projects. This knowledge-gap has hindered the development of a practical tool to support spatial planning related to wave energy projects. Evaluating a site’s capacity for wave energy requires information about various factors including wave power resources, the characteristics of wave energy conversion devices, cost-effectiveness, constraints on siting of energy conversion facilities, and compatibility with other human uses or ecosystem attributes. Marine spatial planning, a nascent effort in North America, is a process in which planners consider the interactions among and cumulative impacts of human activities in coastal and ocean spaces [8]. Efficient marine spatial planning for wave energy projects requires a comprehensive framework for synthesizing the aforementioned diverse information.

Estimating wave power resources can help identify energy-rich and sustained resource areas for potential siting. Previous studies have estimated potential wave power at various scales. For example, studies at global and regional scales show that the west coasts of North America (i.e., British Columbia, Washington, Oregon and California) and Europe (i.e., Ireland, Portugal, and Scotland) are prime regions for wave energy projects because of their potential to generate substantial amounts of energy that can be used to meet high demands from adjacent coastal population centers [9], [10], [11]. Studies focused on the local scale have quantified nearshore wave energy resources and identified wave energy hot spots [12], [13].

Many different types of wave energy conversion devices are available to capture energy from waves, and different technologies vary in how much energy can be harvested as a function of local wave conditions. For example, attenuator-type devices (e.g., Pelamis, developed by Pelamis Wave Power) work more efficiently in conditions typified by the region offshore of Ireland and Scotland [9], where wave heights are high. In contrast, terminator-type devices (e.g., the oscillating water column device from Energetech) work more efficiently along the west coast of North America [14], where waves with longer periods (e.g., swell) dominate.

In reality, efficient siting of a wave energy conversion facility is dictated not only by the potential harvestable energy, but also by revenue and costs associated with constructing and operating the facility. Economic valuation of harvestable wave energy facilitates the evaluation of potential trade-offs between locating a facility in a particular location for energy and the costs of installing, maintaining, and operating the facility at that location. Although methods to harvest wave energy are established [9], [10], [14], [15], most estimates of harvestable wave energy do not provide spatially-explicit information to evaluate a site’s capacity for wave energy generation or the associated costs.

We developed a freely-available decision-support tool capable of 1) providing spatially-explicit information for siting wave energy conversion facilities and 2) helping decision-makers tackle challenges for integrated coastal and marine spatial planning related to wave energy projects. First, we developed the Wave Energy Model as a component of the Integrated Valuation of Ecosystem Services and Trade-offs (InVEST) tool [16], [17], [18]. The wave energy model uses the ecosystem services framework proposed by Tallis et al. [19] and consists of three parts: 1) assessment of potential wave power based on wave conditions (“supply metrics”), 2) quantification of harvestable energy using technology-specific information about a wave energy conversion device (“service metrics”), and 3) assessment of the economic value of a wave energy conversion facility over its life span as a capital investment (“value metrics”). Second, we conducted a compatibility analysis to identify where wave energy conversion facilities and existing marine uses are most compatible. InVEST is composed of a suite of ecosystem service models including the wave energy model described here. The tool is being used for many other coastal and marine spatial planning processes [18] and can be used to support to marine spatial planning related to various ocean renewable energy projects. InVEST is freely available at http://www.naturalcapitalproject.org/InVEST.html (Accessed 2012 October 10).

**Figure 1:**
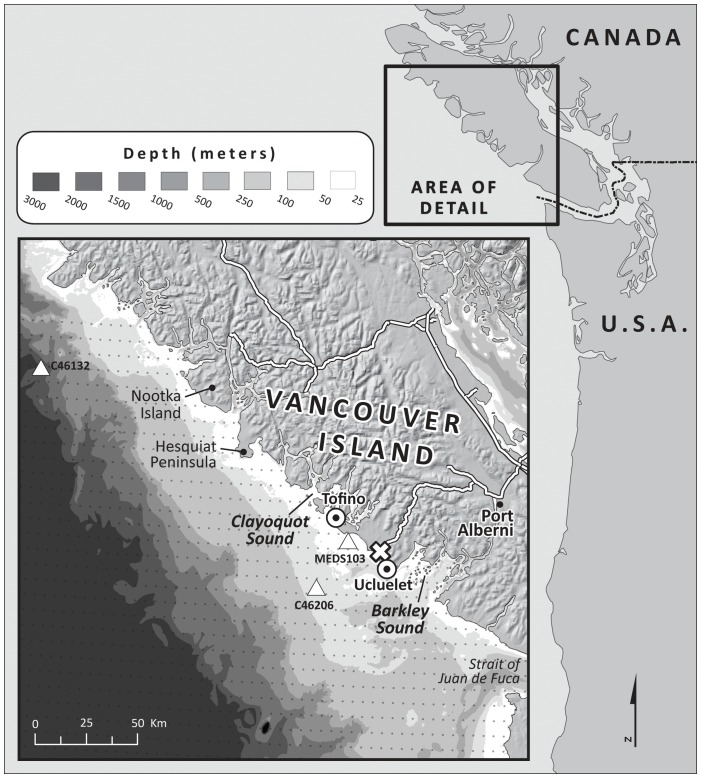
A map of the west coast of Vancouver Island showing three wave buoy stations (Δ), WAVEWATCH III grid points (•), and existing transmission lines (black and white line on the Island) connected to Vancouver Island, British Columbia, Canada. Underwater transmission cable landing points (⊙) are located in Tofino and Ucluelet. Power grid connection point (empty X) is located in Ucluelet.

We applied the wave energy model and conducted the compatibility analysis in a region off the west coast of Vancouver Island (Fig. 1), British Columbia, Canada. Decision-makers and stakeholders on the Island are currently engaged in a marine spatial planning process that includes consideration of renewable energy sources to reduce dependence on mainland Canada for energy [10], [20]. Our modeling on Vancouver Island aims to inform this process as communities on the Island begin to weigh myriad options for future use of their coastal and marine resources. The analysis we present here illustrates how a spatially-explicit estimation of economic returns from wave energy conversion and exploration of the compatibility of promising energy sites with existing uses can help decision-makers and stakeholders decide where to install devices to maximize value from wave energy while minimizing potential conflict with existing uses of coastal and marine ecosystems.

## Methods

### Assessment of Potential Wave Power

The wave energy model estimates potential wave power to identify energy rich areas of the ocean. The wave power transmitted by ocean waves at a certain location can be approximated as [21].
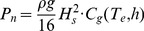
(1)where, *P_n_* is wave power (kW m^−1^), *ρ* is sea water density (1,028 kg m^−3^), *g* is gravitational acceleration (9.8 m s^−2^), *H_s_* is significant wave height, *T_e_* is wave energy period (sec), *h* is water depth and *C_g_* is wave group velocity (m s^−1^ ). *C_g_* can be estimated as
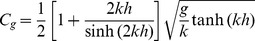
(2)where the wave number *k* is calculated using a dispersion relationship expressed as a function of *h* and wave frequency *w* (*w* = 2*π*/*T_e_*):



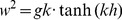
(3)The measured wave period is rarely expressed as *T_e_*, rather, it is often specified as peak wave period, *T_p_*. Therefore, the peak energy period is estimated as *T_e_* = *α⋅T_p_*, where *α* determines the shape of a wave spectrum. In this study, we used *α* = 0.90, which was used in a previous estimation of global wave power resources [21] and in wave power estimates off the west coast of Canada [10], [12].

### Quantification of Harvestable Wave Energy

The amount of energy harvestable from waves in a particular location depends upon wave conditions and the characteristics of wave energy conversion devices [9], [10], [14], [15]. The wave energy model quantifies harvestable wave energy, *WE*, for each sea-state bin (the general condition of the ocean surface) characterized by *H_s_* and *T_p_* as
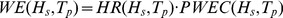
(4)where *HR* (hr yr^−1^) indicates occurrence of hours of each sea-state bin, and *PWEC* (kW) indicates wave energy absorption performance of a wave energy conversion device at each sea-state bin. The annual harvestable wave energy (kWh yr^−1^) per wave energy conversion device in a location is calculated by summing the harvestable wave energy in all sea-state bins at that location.

We conducted a literature review of wave energy conversion devices and prepared wave energy absorption performance tables for several wave energy conversion devices that have undergone full-scale in-situ testing and verification. Currently, the wave energy model includes performance tables for Pelamis [15], [22], Energetech-Terminator [14], AquaBuOY [10], and WaveDragon [10].

### Assessment of the Economic Value of a Wave Energy Conversion Facility

To identify the offshore areas that are most suitable for wave energy development, we use a simple capital investment framework that combines estimates of annual revenue (*R_t_*), capital and construction costs (*C_0_*), and annual operation and maintenance costs (*C_t_*). We assume the wave energy facility has a lifetime of *T* years. To discount the value of future benefits and costs, we use a discount rate (*i*) to compute the net present value (*NPV*) of a wave energy conversion facility:
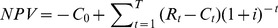
(5)Annual revenue (*R_t_*) is computed as the product of the price of electricity per kWh and annual harvestable wave energy in kWh. Both the discount rate and the wholesale price of electricity are user-defined inputs. The discount rate reflects the opportunity cost of what an individual or firm could obtain with those same funds in alternative investments and can be adjusted by users as appropriate for their location and project. We assume that initial costs for acquiring and installing the wave energy devices are incurred immediately (*t* = 0). These initial costs include: 1) capital costs per installed kW, which is device dependent, 2) the cost of mooring lines, 3) costs of underwater transmission lines, and 4) costs of overland transmission lines. After these initial costs, there are annual operating and maintenance costs for operating the facility that are a function of the size of the wave energy facility. We do not consider the costs of additional land-based infrastructure that may be required to connect an offshore facility to the grid, the costs of permitting or financing a wave energy project, or the salvage value of materials used in a project at the end of the project’s lifespan. Our estimates thus provide a lower bound on the costs of an actual installation. Costs estimates for different wave energy conversion devices were derived from [10] and converted from 2006 CAD to 2009 USD. We use real values for revenues and costs, which do not include any future inflation, and a real discount rate, which similarly does not include an inflation adjustment. This approach is mathematically equivalent to including estimates of future inflation in revenues, costs, and the discount rate [23] but avoids the necessity of developing such estimates. Because the costs of transmission lines depend on the distance of the facility to the nearest grid connection point, the wave energy *NPV* calculation includes the trade-off between locating a facility in nearshore areas where installation costs are lower but the wave power is also lower, resulting in less harvestable energy.

This approach provides a simple mechanism for quickly evaluating offshore sites in terms of the financial viability or *NPV*. Under this simple capital investment framework, if the sum of discounted revenues exceeds the sum of discounted costs, the site is judged to be financially feasible for development. Accordingly, the criterion we use to evaluate whether a site is potentially suitable for wave energy development is a positive *NPV*.

### Identification of Potential Compatibility with Existing Marine Uses

To identify areas that are potentially highly suitable for wave energy conversion projects and are less likely to compromise existing uses, we conducted a simple spatial compatibility analysis. The compatibility analysis shows areas where the existing uses co-occur and are therefore likely to generate the most discussion and, possibly, need for consensus between multiple sectors if spatial co-occurrence produces detrimental effects to existing uses. However, it does not include any judgment of whether the addition of a wave energy conversion facility is likely to be beneficial or detrimental to individual existing uses in a process-based manner.

First, we compile spatially-explicit data about existing activities that occur in nearshore and offshore areas under consideration for wave energy development. Using maps derived from these data, we then determine which human activities co-occur spatially with areas of positive wave energy *NPV*. When the value of an existing human use can be expressed in monetary terms, a more direct comparison of total economic value across existing uses to wave energy *NPV* can be made. In these cases, projecting future revenue and cost streams for existing uses is problematic, and so we converted the wave energy *NPV* to an annualized value [24]:
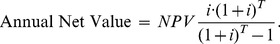
(6)The annual net value is the constant amount, discounted and summed over the *T*-year life of the wave energy facility that makes that sum equal to *NPV*. We then compare this annual wave energy value to a representative annual value of the existing use for which economic data are available.

Lastly, we demonstrate how a comparison between annual net values of existing uses and potential wave energy projects informs policy-making by mapping spatial compatibilities between the two. For each grid cell on a 1 km^2^ gridded seascape, we map the difference between wave energy and the annual net values for existing uses relative to the maximum difference across all grid cells. We also explore how results change by weighting values from existing uses higher than those from potential wave energy projects. We use weighting as a method to account for benefits of existing uses that may not be captured in monetary terms in annual net value (e.g., contribution of fishing to local communities, value people place on local seafood harvest, or enjoyment derived from fishing). Compatible areas indicate where the placement of wave energy conversion facilities is likely to generate the least potential conflict with existing uses. Areas of high compatibility are those that have high annual net value for potential wave energy, but are not used heavily for human activities. In these locations, installation of a wave energy conversion facility could, potentially, be compatible with existing uses. Low compatibility areas are those where the value of existing uses is higher than that of wave energy, indicating that conflict is likely to exist if wave energy facilities are proposed for these areas. Cells with wave energy *NPV* ≤0, indicating net loss, are excluded from the analysis under the assumption that wave energy facilities would not be sited in those locations.

### West Coast of Vancouver Island Application

#### Site Description

The study area, on the west coast of Vancouver Island, Canada, extends from Nootka Island in the North to the terminus of the Strait of Juan de Fuca in the South; the offshore boundary extends 100 km from the shore (Fig. 1). Approximately 40,000 people live along the west coast of the Island, and they are concentrated in the Clayoquot and Barkley Sound regions, in the towns of Tofino and Ucluelet. Currently, more than 60% of the total energy demand on the Island is supplied from mainland Canada [25]. To reduce dependence on mainland Canada for energy, stakeholders and decision-makers are exploring options for alternative and renewable sources of energy [10], [20]. Local wave energy harvest is one option under consideration.

Fishing and recreational industries contribute substantially to local communities on the Island [26]. Salmonids, including Pacific salmon, steelhead, and sea-run cutthroat, and groundfish, particularly halibut, have historically sustained commercial and recreational fisheries in the region. The coastal and marine ecosystems of the region, some of which are in parks and ecological reserves, provide diverse recreational opportunities, including whale watching and kayaking. Siting wave energy conversion facilities nearshore or offshore of Vancouver Island could influence existing marine and coastal activities and affect local communities. Our application of the wave energy model and the compatibility analysis to Vancouver Island can help inform an ongoing marine spatial planning process as stakeholders and decision-makers begin to weigh various options for future use of their coastal and marine resources.

An integrated regional marine spatial planning process is underway on the west coast of Vancouver Island. This process is being led by the West Coast Aquatic Management Board (WCA), a co-management agency with representatives from Federal, Provincial, local and First Nation governments, as well as prominent industries in the region. Through proactive and extensive stakeholder engagement, WCA has convened these diverse stakeholders to work with marine planners to articulate and evaluate several options for the future of their ocean space, such as promoting ocean renewable energy resources. When planning for new ocean ventures such as renewable energy facilities, conflicts may arise among stakeholders because some groups will benefit from the new additions, while others may be adversely affected through loss of access or diffuse negative environmental consequences. To facilitate the resolution of these conflicts, WCA has fostered constructive dialogue among diverse groups of people. They have convened community meetings, used interactive mapping exercises, interviews and a variety of analytical tools, including InVEST. Spatially-explicit ecosystem service models such as InVEST, have demonstrated how the flow of benefits to each stakeholder group will change under alternative scenarios, and this has enabled more transparent, honest discussion of trade-offs [18].

#### Wave input data

We used wave input data to estimate potential wave power and harvestable energy. The observed wave data came from three buoys near the Island [27] (Fig. 1 and Table 1). Buoy-C46132 and C46206 have operated since 1994 and 1998 and provide hourly wave data at 2040 m and 73 m depth, respectively. Buoy-MEDS103 is located at 40 m depth and provides three-hour interval wave data from 1970 to 1998. Although wave buoy data provide the most accurate wave information, their spatial coverage is relatively low (e.g., note that only three wave buoys are available along the entire the west coast Vancouver Island; Fig. 1). Results from a wave model can provide useful, finer-scale wave information as long as model outputs correspond well with observations.

**Table 1:** Wave buoy stations off the west coast of Vancouver Island, Canada.

Wave Buoy Station	Depth (m)	Latitude (deg)	Longitude (deg)	Duration (yr)	Mean Relative Error (%)	
					*H_s_*	*T_p_*
C46132	2040	49.73	−127.92	1994–2010	7	3
C46206	73	48.83	−126.00	1988–2010	14	3
MEDS103	40	49.73	−127.92	1970–1998	6	4

Mean relative error (%) indicates WAVEWATCH III model and data comparison results for significant wave height (*H_s_*) and peak wave period (*T_p_*).

To improve the spatial resolution of wave data necessary to run the wave energy model for Vancouver Island, we used WAVEWATCH III model hindcast reanalysis results [28], [29]. These results were obtained from a 4-minute regional grid system developed for the US West Coast (Fig. 1). Five years of wave data (February 2005–January 2010) at a three-hour interval were used as input for the wave energy model. We calculated the average number of total annual hours that all sea-states, organized into bins, occurred in a particular gridded location (4 minute grid cells) using the five-year WAVEWATCH III model results.

#### Economic assessment of potential wave energy conversion facilities

After approximating potential wave power off of the west coast of Vancouver Island, we quantified the harvestable energy that could be captured by a Pelamis attenuator-type wave energy conversion device. We chose Pelamis for this example because it is the device in the most advanced stage of development [2], [9]. Pelamis does not operate under extreme wave conditions (i.e., *H_s_* >10m and *T_p_*>20 sec) [15] and we excluded all extreme events from harvestable energy calculation. Therefore the harvested wave energy generated annual revenue only when waves were suitable for energy harvest.

For the economic valuation of a wave energy conversion facility, we assumed each facility consisted of 25 Pelamis devices with a 750 kW power rating (i.e., installed energy capacity is 19 MW) and calculated *NPV* over a 25-yr life-span of the facility. The lifespan of wave energy devices ranges from 15–20 years and is highly speculative. We chose to evaluate the project over a 25-year lifespan and assume no significant replacement costs in that time periods. We generated a *NPV* map for the study area by calculating *NPV* assuming placement of a single wave energy conversion facility in each 1 km^2^ grid cell. We placed two potential landing points of underwater transmission cables in Tofino and Ucluelet (Fig. 1), which represent connections to an existing grid connection point in Ucluelet. For this application, we used the annual operating and maintenance costs from [10] and converted all values to 2009 USD. Their estimate is based on the assumption that annual operating and maintenance costs represent approximately 2% of capital expenditures for devices operating at 20% capacity on average. The underwater transmission cable cost per km depends on the capacity of transmission cable used. Dalton et al. [9] reflect large variability in these costs ranging from USD 500,000 to over USD 5,000,000 km^−1^ as a function of wave energy conversion power output. In this analysis, we used USD 1,664,000 km^−1^ for underwater transmission cable cost, which is appropriate for the installed energy capacity (i.e., 19MW) on the west coast of Vancouver Island. In other applications, these costs can be adjusted to best reflect actual costs for any particular area. The cost of electricity in the model is based on feed-in tariffs for electricity produced by wave energy, which ranges from USD 0.11–0.36 kWh^−1^ [9]. In this study, we used the median of the costs, USD 0.235 kWh^−1^. See Table 2 for all the parameters for the *NPV* calculation.

**Table 2:** Economic parameters for net present value (*NPV*) assessment for the Pelamis wave energy conversion device.

NAME	VALUE
Maximum capacity of device (kW)	750
Capital cost per installed ($ kW^−1^)	3,671
Cost of mooring lines ($ m^−1^)	20
Cost of underwater transmission cable ($ km^−1^)	1,664,000
Cost of overland transmission cable ($ km^−1^)	64,499
Operating & maintenance cost ($ kWh^−1^)	0.042
Price of electricity ($ kWh^−1^)	0.235
Discount rate	0.08

#### Existing marine uses compatibility analysis

To identify marine uses that overlap with areas of positive *NPV* for wave energy, we performed two types of analyses using data on: 1) human uses and ecological characteristics of the west coast of Vancouver Island and 2) economic value of commercial fisheries. The first allowed us to conduct comprehensive analysis of spatial overlap, which is appropriate for the broad scope of marine spatial planning in the region. The second data source supported our more in-depth analysis of economic trade-offs between commercial fisheries and potential wave energy facilities, which we used to demonstrate the types of quantitative analyses that can provide a more complete evaluation of trade-offs.

For the first analysis, we used spatially-explicit data on diverse human uses and ecological characteristics of the west coast of Vancouver Island from the British Columbia Marine Conservation Analysis (BCMCA) project [30]. As part of BCMCA’s effort to identify areas that both had high ecological value and were important for human uses, they compiled data on ecological characteristics and seven types of uses in the marine environment, which we aggregated into the following five categories (Table 3): 1) ecological characteristics, 2) shipping and transport, 3) tenures & offshore energy, 4) tourism & recreation, and 5) commercial fisheries. BCMCA summarized information for each category. The shipping & transport category, for example, contains spatially-explicit information for ferry routes, ferry terminals, and densities of cruise, carrier, fishing, tanker and tug vessels. Details for each category are shown in Table 3. The data are at a 2 km^2^ spatial resolution where each grid cell is a tally of the number of human uses or ecological characteristics within that category. BCMCA made the summary data layers publicly available for use in GIS. We used these maps, but limited the spatial extent to the area used for the wave energy analysis off of the west coast, instead of the entirety of British Columbia. We overlaid BCMCA’s maps for each category with areas of positive wave energy *NPV*. In this area of overlap, we recorded the minimum, maximum, and median number of uses or ecological characteristics that occurred across all grid cells in the area of overlap. We report each of these values by quartiles, to facilitate comparison across categories. Quartiles reported as relatively very low, low, moderate, or high were calculated from the range from 1 use to the maximum number of uses in the category for the area off of the west coast of the Island.

**Table 3:** Degree of spatial overlap between areas of positive annual net present value from wave energy facilities and five categories of existing uses and ecological characteristics.

Category	Overlap (median)	Overlap range	Human uses or ecological characteristics in the category
1. ecological characteristics	very low	very low to low	fish and invertebrates; marine birds; marine mammals; marine plants; physical characteristics
2. shipping and transport	moderate	low to high	ferry routes and terminals; density of cruise, carrier, fishing, tanker, and tug vessels; tow boat reserves
3. tenures and offshore energy	very low	very low	aquaculture; log handling & storage; residential marine; commercial & industrial uses; utilities; offshore petroleum; oil and gas prospectivity
4. tourism and recreation	very low	very low to low	anchorages; coastal campsites and kayak use sites; commercial recreation tenures; environmental tenures; marinas and coastal facilities; protected areas; recreational boating routes; sea kayaking routes; SCUBA dive sites
5. commercial fisheries	low	low to high	Dungeness crab; geoduck; krill; shrimp; prawn; sea cucumber; sea urchin; groundfish trawl; rockfish hook and line; schedule II fishery; halibut; sablefish; herring roe; sardines; salmon

Overlap is expressed by quartiles (very low, low, moderate and high) of the median and range (minimum to maximum) of the number of existing uses in 2 km^2^ cells (see Figure 6) that overlap with areas of positive net present value for wave energy. See text for further explanation and consult [30] for the full list for each category.

**Figure 2:**
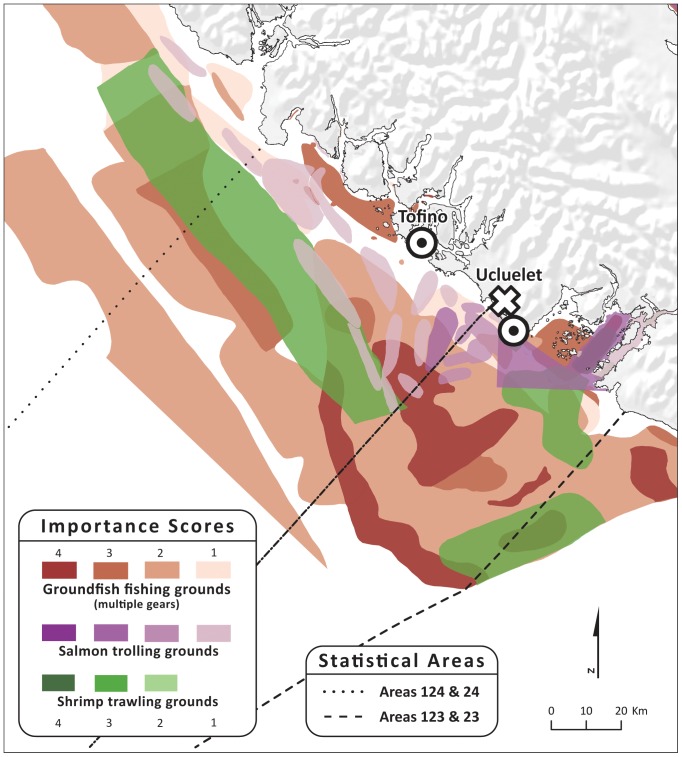
Department of Fisheries and Oceans Canada statistical areas and fishing grounds for groundfish fishing (multiple gears), salmon trolling, and shrimp trawling. Fishing grounds are shaded by their importance (scale: 1–4); high values indicate more important grounds.

Commercial fisheries data were not publicly available, so for the second spatial compatibility analysis, we used maps (Fig. 2) generated by the Province of British Columbia for three commercial fishing fleets: 1) groundfish trawl, longline and handline fishing, 2) Pacific salmon troll fishing, and 3) shrimp trawl fishing. These maps represent data collected by the Department of Fisheries and Oceans Canada from 1993–1996 through interviews with fisheries officers on the west coast of Vancouver Island [31]. Fisheries officers were asked to identify areas used by specific fishing fleets and to qualitatively assess the importance of each fishing area relative to all other fishing areas used by a specific fleet. To facilitate comparison between existing fishing areas and wave energy, the polygons that represent the three commercial fishing fleets were mapped on the same 1 km^2^ grid used for the wave energy *NPV* calculation. While these data are outdated, they are the most comprehensive data available and serve to illustrate the method.

We analyzed the data for the 3 commercial fisheries that overlapped spatially with areas of positive wave energy *NPV,* because we had enough data to characterize the economic value of individual fishing grounds. Data on annual harvest revenues (i.e., landed value) are available [32], [33] for large areas known as “statistical areas” used for government management of the fisheries by the Department of Fisheries and Oceans (Fig. 2). We combined data for statistical areas 23 (inshore) and 123 (offshore), and 24 (inshore) and 124 (offshore), which were the areas that overlapped with areas of positive wave energy *NPV*. Fishing is not uniformly distributed within these statistical areas, however, so overlaying them on areas of positive wave energy *NPV* would give an overly pessimistic view of the potential compatibility of the two activities. We thus used information on the location and quality of individual fishing grounds within each statistical area to refine the spatial location and value of commercial fishing [31]. These data included information on the importance of individual fishing grounds for each fleet, on a scale from 1 (very low) to 4 (high) (Fig. 2). We used these numerical scores to apportion the average 1982–2001 harvest revenue (converted to 2009 USD and inflation adjusted) for a statistical area to individual fishing grounds by using the scores as relative weights. For example, a fishing ground rated 2 was assigned twice as much revenue per km^2^ as a fishing ground rated 1, and so forth. Relative weights were used linearly in the absence of any information to indicate that we should scale them otherwise (e.g., logarithmically). We assumed areas that were not rated did not support any harvest and so we assigned them zero harvest revenue. We then converted the gross economic value of each fishing ground to a net value by assuming that costs account for 40% of gross revenue, which falls within the range of costs observed in various fisheries operating off the central California coast [34]. The spatial areas identified as individual fishing grounds were converted to 1 km^2^ grid cells, which facilitated their comparison to the areas used to evaluate wave energy *NPV* (also 1 km^2^ grid cells).

Finally, we conducted the compatibility analysis under the assumptions that annual net values for existing uses and potential wave energy conversion facilities were 1) equally weighted and 2) that fishing values were 50 times the weight of wave energy values. Weighting can account for unmeasured benefits of existing uses that have a monetary metric, as we are doing for commercial fishing. Weighting can also be used to account for benefits of the other uses, in that the (inverse of the) weight represents the proportion of all benefits that are accounted for by the measured, monetary benefits of commercial fishing. Thus, a weight of 50 is equivalent to expressing that commercial fishery “profits” account for two percent of all benefits from all other non-wave energy uses. We considered these weights broad enough to capture a range of preferences that may be expressed by policymakers.

## Results

### Wave Condition on the West Coast of Vancouver Island

**Figure 3:**
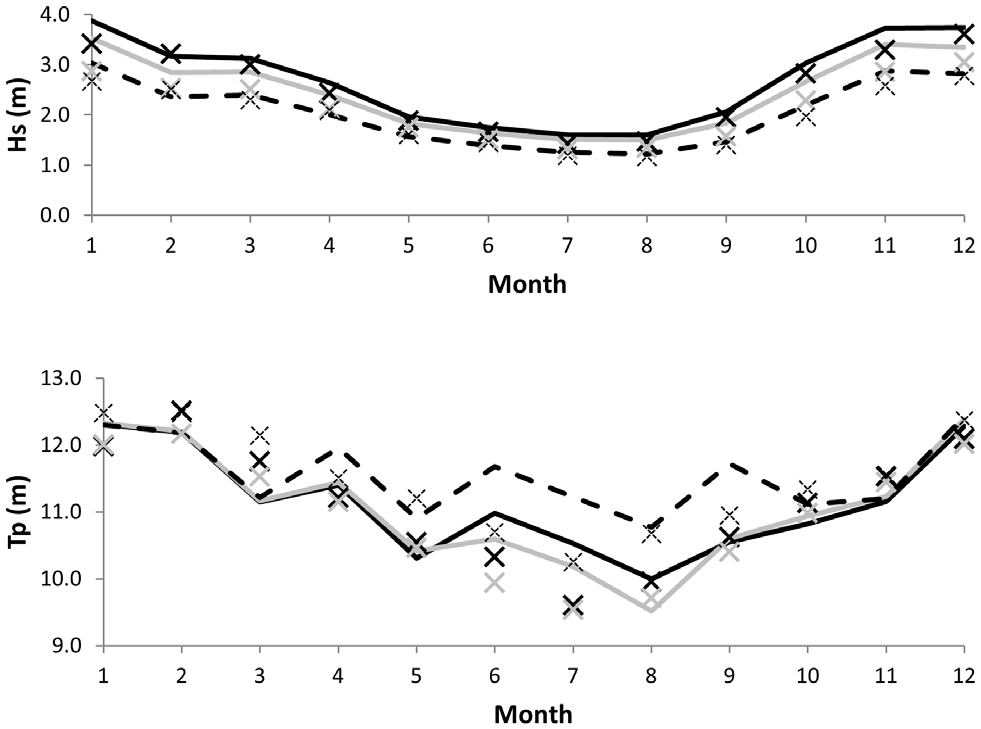
Comparison of wave buoy data (X) and WAVEWATCH III model hindcast reanalysis results (lines) for significant wave height (*H_s_*) and peak wave period (*T_p_*) monthly averages at three wave buoy stations: C46132 (solid black), C46206 (light gray), and MEDS103 (dashed).

Our analysis of buoy data showed that wave conditions became stronger from nearshore (Buoy-MEDS103) to offshore (Buoy-C46132) and that there was significant seasonal variation (Fig. 3). Average winter wave height (November-January) was approximately twice that in summer (June-August), with intermediate wave heights in spring and fall. Wave period had a similar seasonal pattern, with longer wave period in winter and shorter period in summer.

WAVEWATCH III model results showed good agreement with wave buoy data (Fig. 3). Relative mean error was 6–14% for *H_s_* and 3–4% for *T_p_* (Table 1), indicating that wave input based on WAVEWATCH III model results represented the observed long-term average wave conditions well. The wave input also represented the observed seasonal patterns well, with larger *H_s_* and longer *T_p_* in winter and smaller *H_s_* and shorter *T_p_* in summer.

### Potential Wave Power and Harvestable Wave Energy

**Figure 4:**
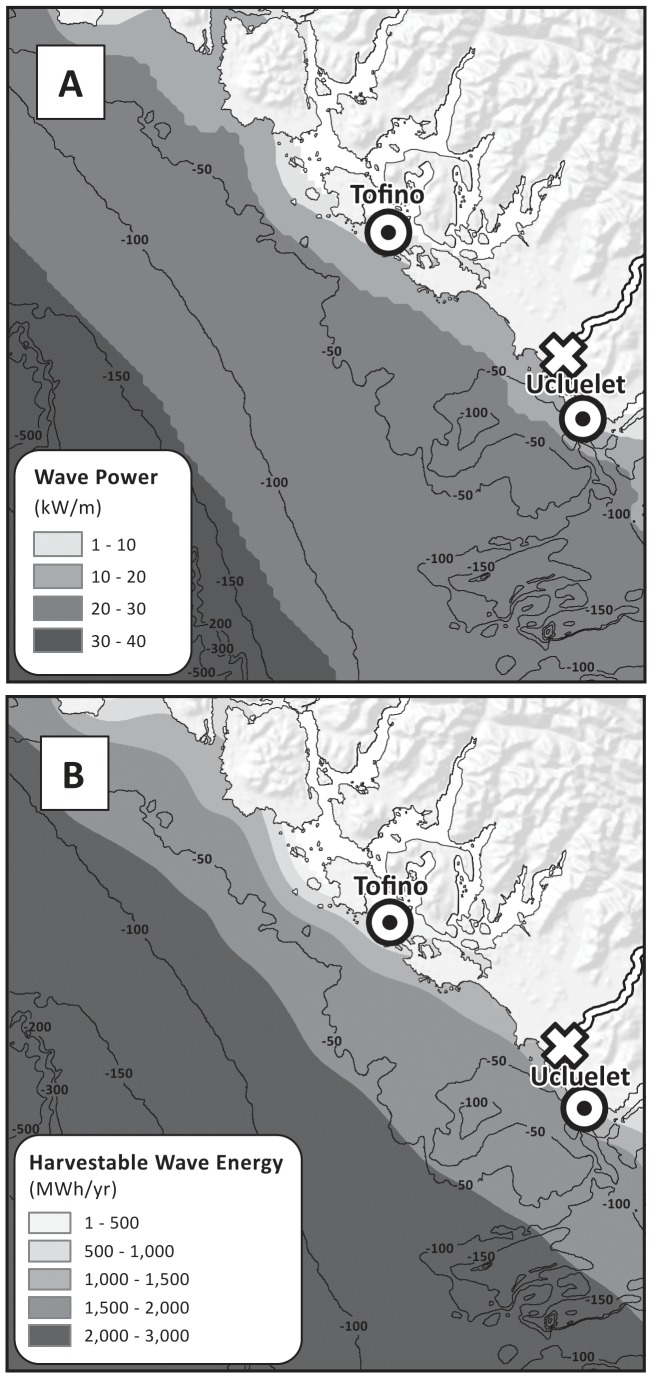
Potential wave power (kW m^−1^) (A) and harvested wave energy (MWh yr^−1^) (B) using a Pelamis wave energy conversion device with 750 kW power rate. Black contour lines indicate water depth in meters. Underwater transmission cable landing points (⊙) are located in Tofino and Ucluelet. Power grid connection point (empty X) is located in Ucluelet.

Annual mean wave power was greater than 10 kW m^−1^ in most areas (Fig. 4A), and gradually increased offshore to maximum of 30–40 kW m^−1^ at depths greater than 150 m. Higher wave power (30 kW h^−1^ power contour) was closer to shore in the north (∼38 km from Tofino) than in the south (∼47 km from Ucluelet). Wave power varied seasonally (not shown), with maximum wave power (30–80 kW m^−1^) in winter and substantially less (<20 kW m^−1^) in summer.

The overall patterns of the harvestable wave energy were similar to those of potential wave power (Fig. 4B). A Pelamis device located at the 50–70 m depth contour (the optimal depth range for Pelamis) would produce approximately 1,500–2,500 MWh yr^−1^ per device, which, assuming 15 MWh yr^−1^ energy use per household on Vancouver Island [25], is enough energy to support 100–167 households. Model outputs also showed that harvested wave energy by Pelamis devices displayed a strong seasonal pattern (not shown), with maximum energy capture in winter and minimum in summer.

### Economic Value of a Wave Energy Conversion Facility

**Figure 5:**
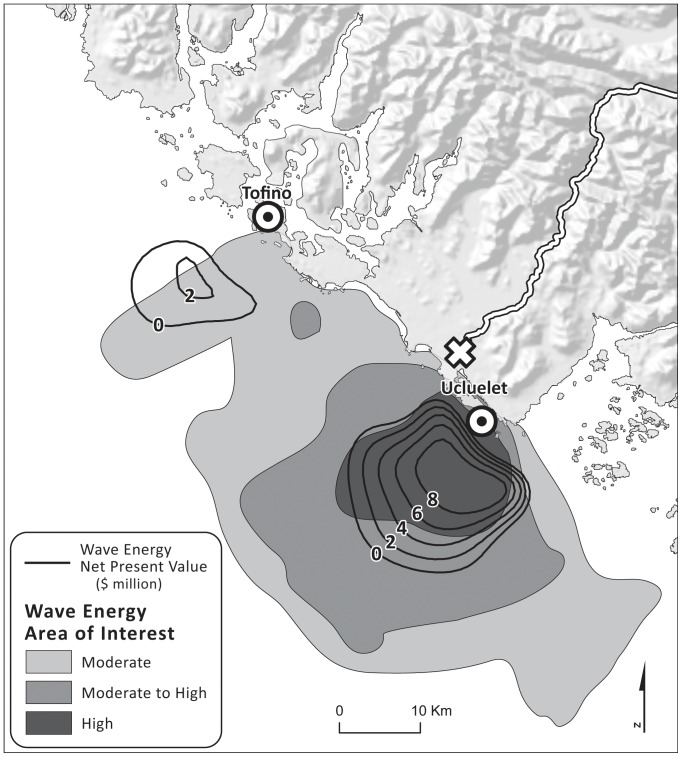
Wave energy net present value in million USD (black contour lines) over a 25-year life-span of wave energy conversion facilities and wave energy areas of interest (gray contours) modified from British Columbia Marine Conservation Analysis Atlas [20], [35]. Underwater transmission cable landing points (?) are located in Tofino and Ucluelet. Power grid connection point (empty X) is located in Ucluelet.

Negative *NPV* occurred in nearshore areas due to the poor wave conditions, indicating a net loss from wave energy conversion facilities (Fig. 5). Positive *NPV* started from 2 and 9 km offshore from the two underwater transmission cable landing points in Ucluelet and Tofino, respectively. The *NPV* increased with distance from the coast and its peak (highest 20% of values, $8 - 10M) occurred 5–11 km from the landing points in Ucluelet, which are the best potential sites for a wave energy conversion facility, considering only the economics of wave energy capture. *NPV* decreased further offshore because of increasing underwater cable costs, and shifted to negative value again at approximately 20 km from the landing points of the underwater transmission cables.

### Compatibility with Existing Marine Uses

**Figure 6:**
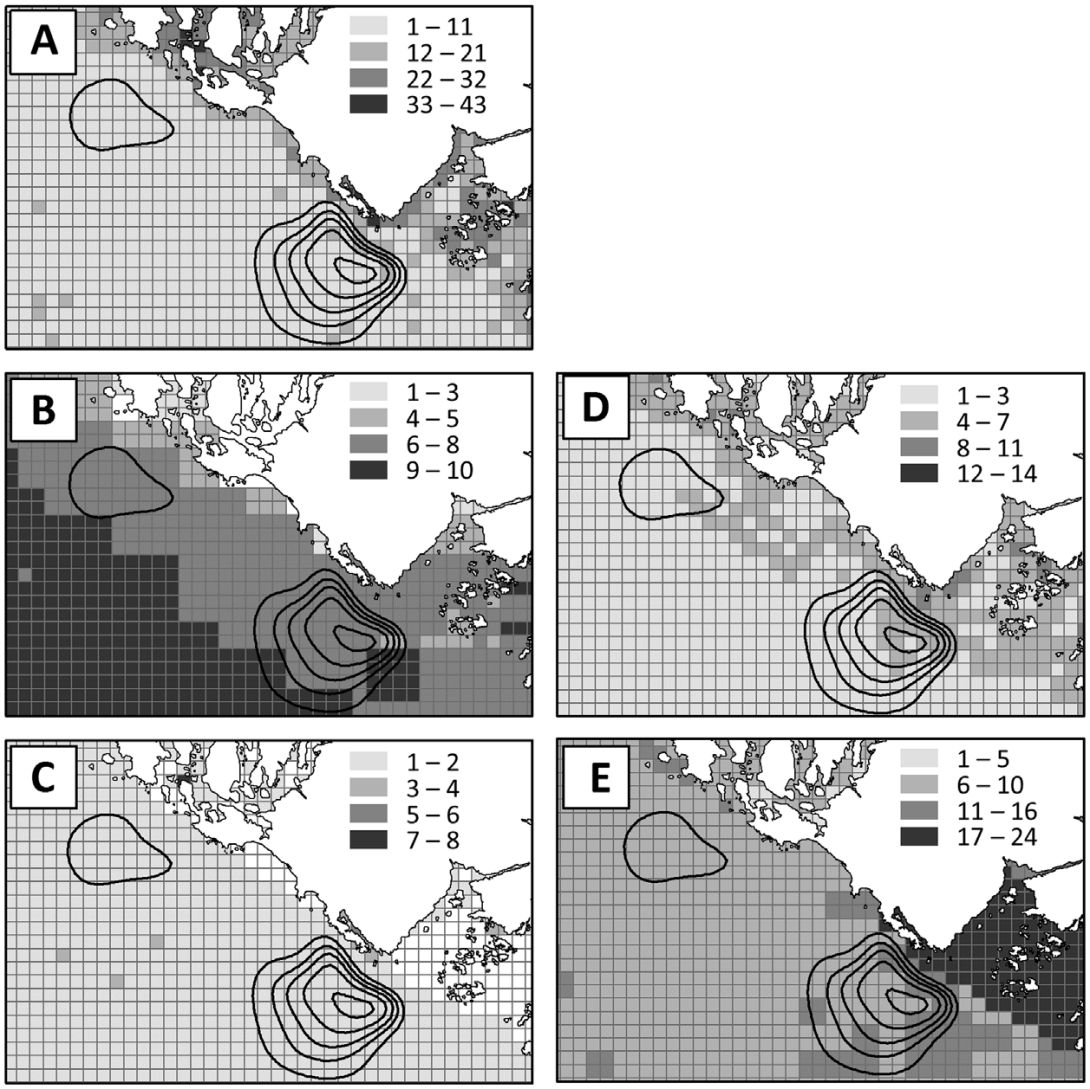
Overlap between areas of positive annual net present value over a 25-year life-span of wave energy conversion facilities (black contour lines; value in million USD) and five categories of existing uses or ecological characteristics (gray grids): A) ecological characteristics, B) shipping and transport, C) tenures and offshore energy, D) tourism and recreation, and E) commercial fisheries. The inset legend indicates the number of existing uses or ecological characteristics in the category that occur in a grid cell.

Across the 5 categories (ecological characteristics, shipping and transport, tenures and offshore energy, tourism and recreation (includes sportfishing), and commercial fisheries) areas with positive wave energy *NPV* overlapped at least one use or ecological characteristic in each category. The highest median overlap occurred with shipping and transport, and the second with commercial fisheries uses (Fig. 6 and Table 3). Spatially, areas of higher overlap with shipping and transport and commercial fisheries were located offshore of Ucluelet. The median overlap with the other categories was “very low” for ecological characteristics, tenures and offshore energy, and tourism and recreation.

**Figure 7:**
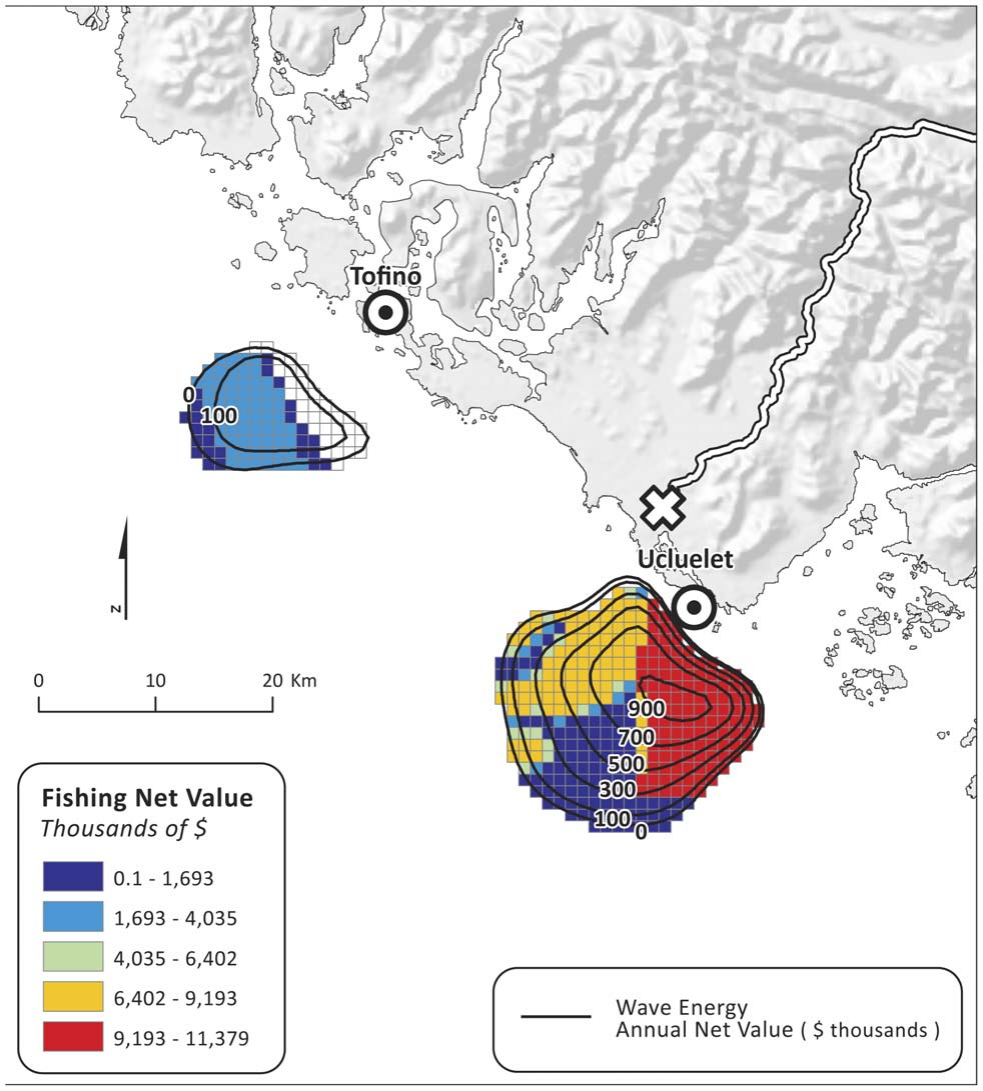
Net value (USD) of three commercial fishing layers and wave energy annual net value (thousand USD; black contour lines). Underwater transmission cable landing points (?) are located in Tofino and Ucluelet. Power grid connection point (empty X) is located in Ucluelet.

**Figure 8:**
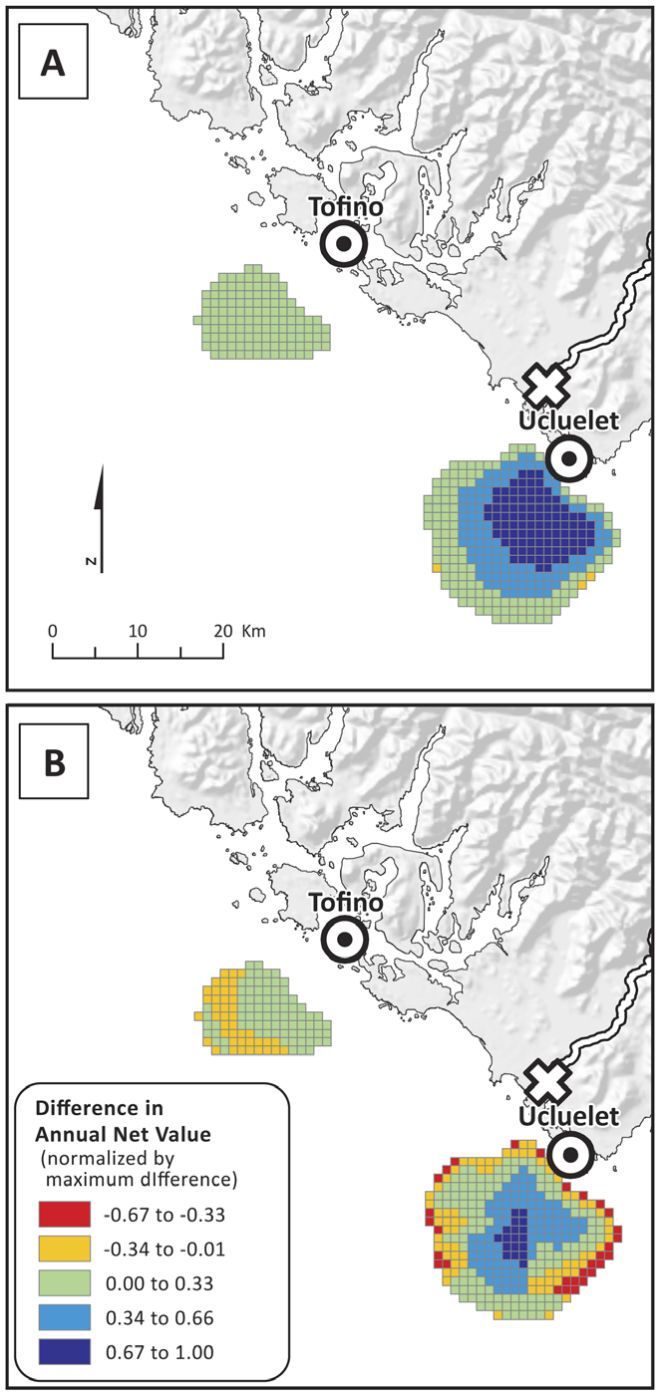
Compatibility analysis between wave energy and fishing annual net values, where A) values are given equal weight and B) fishing values are weighted 50 times that of wave energy values. Underwater transmission cable landing points (⊙) are located in Tofino and Ucluelet. Power grid connection point (empty X) is located in Ucluelet.

The annual net value per grid cell for the three fishing fleets combined had a maximum of $11,379 km^−2^ and was highest offshore of Ucluelet (Fig. 7). We mapped spatial compatibilities between the two as the difference (fishing from wave energy) for each grid cell relative to the maximum difference assuming 1) that policymakers give equal weight to values (Fig. 8A) or 2) that policymakers weight values from fishing at 50 times their economic values (to reflect the additional cultural and other values attached to fishing; Fig. 8B). Values close to 1 are considered most compatible. When values are equally weighted (Fig. 8A), all areas of positive annual net value for wave energy are considered compatible with fishing because the difference in values between the two sectors is so large. However, when fishing values are weighted higher than wave energy values (Fig. 8B), the size of the highly compatible area offshore of Ucluelet is smaller.

### Potential Candidates for Wave Energy Conversion Facilities

In a separate effort, industry representatives, scientists, and resource managers identified potential areas for development of the wave energy industry based on expert judgment by those with general knowledge of the distribution of spatial uses in the area [20], [35]. They concluded that areas offshore of Ucluelet (Fig. 5) had the highest potential for wave energy projects based on various factors, including the wave climate, transmission infrastructure, comparability of wave energy projects with other environment or socio-economic values, and physical site characteristics (e.g., substrate, proximity to land). The positive *NPV* map from our model identified economically beneficially areas, which showed a good match with the areas identified by expert opinion as having the highest potential for wave energy conversion projects (Fig. 5). Such a match indicates that the simple framework for economic feasibility analysis captures the main factors considered important by stakeholders for determining potential sites for wave energy projects. In addition, the compatibility analysis further pinpointed potential sites (Fig. 6 and 8) for wave energy projects that have relatively low conflicts with a suite of existing uses as well as provide maximum financial benefits.

## Discussion

Decision-makers and the public are interested in converting wave energy into clean, safe, reliable and affordable renewable energy. With this increasing interest in wave energy, there is a growing need for better-integrated information to help site wave energy facilities to reduce use conflicts. To inform siting decisions, we developed and applied a wave energy model that quantifies potential wave power and harvestable energy and, in addition, evaluates the financial feasibility of a wave energy conversion facility. Also, we conducted an analysis to identify regions of the ocean along the west coast of Vancouver Island where wave energy facilities are likely to be most compatible with existing marine uses. Importantly, we designed our analysis to be flexible and applicable in other locations worldwide, where wave energy is of interest, and we created a tool to facilitate future applications of this approach [16], [18].

We have shown that: 1) it is possible to use spatially-explicit information to explore locations where wave energy facilities might be profitable, 2) simple syntheses of existing ocean uses can further inform the siting of wave energy facilities by exploring potential conflicts with existing uses and 3) high-value sites for wave energy facilities can be found in regions where spatial overlap with existing uses is minimal, thus demonstrating that a comprehensive, cross-sector approach can help identify compatibilities among human activities and lead to win-win situations for arraying ocean uses in coastal regions.

In our application of the wave energy model off the west coast of Vancouver Island, we found that wave conditions provided wave power greater than 10 kW m^−1^, the minimum needed for a commercial scale wave energy project [36]. Harvestable wave energy using an attenuator type device, Pelamis, gradually increased offshore as wave conditions intensified. Harvestable wave energy showed a seasonal pattern, with maximum energy in winter and minimal energy in summer. Since the residential energy demand is highest in winter and lowest in summer [25], wave energy could be an appropriate match for supplying energy to meet the residential electricity demands on the west coast of Vancouver Island.

The location of a wave energy conversion facility is dictated not only by the potential energy availability and harvestable energy. It is important to also consider how energy capture may be offset by costs of installation and maintenance and compatibility with existing human uses. First, the trade-offs between benefits of harvested energy and various costs determine the wave energy *NPV* that indicates net benefits of a facility. We showed that economic hotspots with high *NPV* were much closer to shore than areas with maximum potential wave power and harvestable energy.

These nearshore economic hotspots are likely candidates for wave energy conversion facilities. Further offshore from the hotspots, the high cost of lengthy underwater transmission cables outweighs the benefits from energy harvest, which leads to lower *NPV*. The model results highlight that wave power resources at a wave energy conversion facility site and the distance to the landing point for the underwater transmission cable play a critical role in determining the economic feasibility of a wave energy conversion project. The results also highlight areas where innovation could occur. Reductions in the cost of offshore transmission cables would allow wave energy facilities to tap into areas with higher potential wave energy while simultaneously reducing co-occurrence with many existing human uses that tend to be clustered closer to shore.

Even once the financially feasible area of the oceans in which wave energy facilities might profitably be sited is identified, there are still important considerations to weigh when making siting decisions. Siting wave energy facilities in regions where facilities would be most profitable without consideration of other ocean uses could hamper existing ocean uses, affect the well-being of coastal communities, and cause conflict among stakeholders [5]. Because wave energy conversion facilities may exclude other ocean-based activities, particularly some types of recreation and commercial fishing [37], siting of facilities that will lead to minimal conflict with people who live and work in the focal region involves finding areas with few existing marine uses. We found that all areas with potential for wave energy facilities (i.e., positive wave energy conversion facility *NPV*) overlap with at least one existing human use or important ecological characteristic, and that shipping and transport and commercial fisheries were the mostly commonly co-located activities with these areas. The simple step of overlaying existing and proposed uses helped us identify particular sectors that will need to work together and which sectors or categories of use are likely to be less affected. A similar approach to siting wind-energy facilities along the US Atlantic Coast identified areas where spatial conflicts can be minimized in the presence of energy generation facilities [4]. For complex, multi-sectoral planning processes like the marine spatial planning process underway on the west coast of Vancouver Island, these simple overlay exercises can help stakeholders and decision-makers refine their planning efforts.

The degree to which wave energy is compatible with other sectors will depend on several factors, including how valuable the proposed area is for other uses. In this analysis, we consider areas with highest fishing annual net values to be those that are most important. When mapped with wave energy annualized *NPV*, we see that there are several areas with high wave energy and low fishing annual net values. Our compatibility analyses confirm that, indeed, there are high compatibility areas within high wave energy *NPV* areas, particularly offshore from Ucluelet, even when policymakers give disproportionate weight to fishing as compared to wave energy. Given that trade-offs between wave energy and existing uses are only beginning to be evaluated [38], [39], the compatibility analysis presented here is illustrative as an initial framework for identifying where potential conflicts and trade-offs may arise. This analysis can help decision-makers find profitable areas for wave energy conversion facilities that minimally impact existing ocean uses.

The strength of any conflict will also depend on whether the wave energy conversion facility and existing uses can co-exist spatially or if they are mutually exclusive. The existing uses that overlapped with positive wave energy *NPV* areas in our analysis all may have different degrees of compatibility depending on how the wave energy conversion facilities are operated and the stringency of regulations about conducting activities near the facilities. If fishing vessels are somehow permitted to passively (e.g., long-lining) or actively (e.g., trawling, trolling) fish their gear through an array of wave energy conversion devices, the impact of the installation of a wave energy conversion facility on existing ocean uses may be slight. Conversely, if the array of wave energy conversion devices is installed contingent upon exclusion of other ocean uses such as fishing, activities may be incompatible, and conflicts large. However, if the size of the wave energy conversion facility is small relative to the fishing grounds, fishers may have sufficient substitute fishing grounds to use and the uses may be considered compatible. Because the wave energy industry is still in a nascent phase, there is little research on spatial compatibility with other activities in marine ecosystems [5], [6]. However, the industry is rapidly developing, and with further real-world experience should come gains in our understanding of how the harvesting of wave energy interacts with other marine uses.

Beyond issues of spatial exclusivity, wave energy conversion devices themselves may or may not detract from the capacity for existing uses. For example, wave energy conversion devices can function as fish aggregating devices, or pseudo-habitat that may provide a net benefit for species or ecosystem attributes, which may in turn benefit commercial and recreational fishers [6], [7], [37], [40], [41], [42], [43], [44], [45]. For recreational fishing or whale-watching, however, wave energy conversion devices may be considered visual pollution to tourists who charter boats seeking to catch fish or see whales in what they perceive to be a pristine setting. In addition, the wave energy conversion devices may have halo effects, such as noise that affects marine mammals or disruptions to foraging by seabirds [6], [7], [37], [38], [44], [45]. Further research on the role of wave energy conversion devices as habitat, and local input on consumer preferences can inform assumptions about which spatial overlaps identified in the compatibility analysis are most likely to involve substantive trade-offs and which are cases where co-occurrence of activities is tractable. A clearer understanding of the synergies or exclusivity of existing uses and wave energy conversion facilities can help shape the discussion in areas with low or negative compatibility scores.

Finally, our estimates of the economic value for wave energy and fishing revealed that potential wave energy conversion facilities can generate value that is substantially higher than existing fishing value. If all stakeholders received benefits from both revenue streams and if a choice was to be made between fishing and a wave energy conversion facility, a straightforward comparison between financial values of the two activities may be possible. However, the assumption that the benefits from both activities are distributed equally among all stakeholders is unlikely to be true; these activities benefit different groups and communities. Further, a simple financial comparison belies the diverse values people attach to fishing activities and other benefits from marine ecosystems. For example, fishing contributes substantially to local Vancouver Island communities in numerous cultural and other intangible ways, and so harvest revenues do not fully capture the benefits of the three fisheries. The areas off the coast of Ucluelet have long supported seafood harvest for food, social and ceremonial purposes for the Ucluelet First Nation.

Our analysis of the two types of value serves to motivate this broader discussion about who benefits from each activity. While our estimates of value for wave energy and fishing can be improved (e.g., inclusion of more detailed costs of fishing, variability in wave energy and fish harvest), their comparison is a useful impetus for a broader discussion about beneficiaries and trade-offs. Ultimately, the decision about where to site new wave energy facilities is a decision that affects a whole range of stakeholders in the region: residents of the towns of Tofino and Ucluelet, several First Nations, the commercial fishing and tourism industries, as well as visitors who come to the area from around the world. Mapping and valuing the benefits to each of the groups, and involving these diverse stakeholders in a deliberative decision-making process is a promising way to achieve a successful marine spatial plan for the region.

The compatibility analysis can be improved in several ways. The data used for the commercial fisheries analysis reflect fisheries and other human uses from 1993–1996. More recent data on fishing regulations, fleet activity and the abundances of target species would lead to a more accurate evaluation of current compatibility. Further, our method of identifying the most preferable areas for each fishing fleet – via qualitative importance scores – could be improved with spatially-explicit catch or effort data, neither of which was available to us for this analysis. Also of interest, but unavailable for this research, would be routes used to transit to fishing grounds, as these transit routes could be impacted by new wave energy conversion facilities. The analysis would also be made more robust by including spatially-explicit data on the economic value of other uses (e.g., recreational, shipping) with the commercial activities. Some of these data gaps could be filled through a continued mapping or additional stakeholder outreach processes [46]. The analysis presented here, nonetheless, serves a valuable purpose by illustrating the method and generating productive discussion about where and what types of compatibilities and incompatibilities may exist, and what additional existing uses should be included in the analysis.

Our approach may be useful for decision-makers, stakeholders, and environmental groups seeking to understand the potential for wave energy projects to contribute to renewable energy and to fit into the mix of existing and potential future uses in a particular location. The model’s flexible framework allows it to be applied at local, regional, and global scales to help decision-makers explore alternative sites for wave energy facilities in terms of the benefits from harvesting wave energy, the effects on coastal and ocean ecosystems, and the trade-offs with other human uses. With rapid growth in renewable energy industries and the onset of an era of coastal and marine spatial planning comes a need to quickly and effectively assess where activities can co-exist and where we need to proactively expend effort to minimize potential conflict; the approach presented here is particularly well-suited to meet this need.
